# Neurobiological regulation of eating behavior: Evidence based on non-invasive brain stimulation

**DOI:** 10.1007/s11154-021-09697-3

**Published:** 2021-12-04

**Authors:** Theresa Ester, Stephanie Kullmann

**Affiliations:** 1 Institute for Diabetes Research and Metabolic Diseases of the Helmholtz Center Munich at the University of Tübingen, Tübingen, Germany; 2 German Center of Diabetes Research (DZD), Tübingen, Germany; 3 Department of Internal Medicine, Division of Endocrinology, Diabetology and Nephrology, Ebehard Karls University Tübingen, Tübingen, Germany

**Keywords:** Eating behavior, Neurostimulation, Transcranial direct current stimulation, DLPFC, Food craving, Cognitive control, Obesity

## Abstract

The prefrontal cortex is appreciated as a key neurobiological player in human eating behavior. A special focus is herein dedicated to the dorsolateral prefrontal cortex (DLPFC), which is critically involved in executive function such as cognitive control over eating. Persons with obesity display hypoactivity in this brain area, which is linked to overconsumption and food craving. Contrary to that, higher activity in the DLPFC is associated with successful weight-loss and weight-maintenance. Transcranial direct current stimulation (tDCS) is a non-invasive neurostimulation tool used to enhance self-control and inhibitory control. The number of studies using tDCS to influence eating behavior rapidly increased in the last years. However, the effectiveness of tDCS is still unclear, as studies show mixed results and individual differences were shown to be an important factor in the effectiveness of non-invasive brain stimulation. Here, we describe the current state of research of human studies using tDCS to influence food intake, food craving, subjective feeling of hunger and body weight. Excitatory stimulation of the right DLPFC seems most promising to reduce food cravings to highly palatable food, while other studies provide evidence that stimulating the left DLPFC shows promising effects on weight loss and weight maintenance, especially in multisession approaches. Overall, the reported findings are heterogeneous pointing to large interindividual differences in tDCS responsiveness.

**Supplementary information:**

The online version contains supplementary material available at 10.1007/s11154-021-09697-3.

## Introduction

Obesity, one of the most serious public health problems, has reached epidemic proportions [1–3]. The WHO estimates that the prevalence of overweight has tripled since 1975, leading to more than 1.9 billion overweight and 650 million obese adult people [4]. Overweight and obesity are associated with a variety of diseases such as several cancer types, dementia, depression, cardiovascular disease [5–7] and play a crucial role in the development of type 2 diabetes (T2D) and insulin resistance (IR) [8]. Although obesity and overweight is most commonly caused by a long-term energy imbalance [6], the etiology is multifactorial, including genetic, social, economic, environmental, physiologic and psychological factors [9]. Broadly speaking, the pathophysiology of obesity is complex and still not fully understood. Beyond basic homeostatic mechanisms, the regulation of food intake in humans is established by a core brain network of cognitive control and reward processing pathways (for reviews see [10–13]). In recent years, modern neuroimaging methods, such as functional magnetic resonance imaging (fMRI), have provided new means to investigate key neurobiological determinants of human eating behavior and offer the opportunity to identify novel target structures for interventions. Accordingly, neuroimaging studies suggest that individuals affected by overweight and obesity show dysregulation of the mesolimbic reward and prefrontal cortex (PFC) cognitive control system (for recent review [13]). In the recent years, new treatment modalities have been explored to facilitate behavioral changes that enable successful weight loss. Non-invasive brain stimulation (NIBS) techniques such as transcranial direct current stimulation (tDCS) represent novel tools able to influence neuronal activity [14–20]. Yet, it is still unclear if stimulating specific brain areas, linked to overconsumption, improves food related outcomes on the behavioral level. Despite the growing interest in tDCS as an intervention tool, effects are inconsistent making previous results difficult to replicate [21]. Here we review tDCS studies targeting PFC function to influence food craving and food intake with the aim to broaden our understanding of the underlying neurophysiological mechanisms and modulators responsible for the effect of tDCS on eating behavior. The dorsolateral prefrontal cortex (DLPFC) is a key player in dietary self-control and most commonly targeted by tDCS studies on eating behavior. Therefore, this review will specifically focus on tDCS of the DLPFC.

## DLPFC and its role in eating behavior regulation

The DLPFC is most commonly associated with executive functions, such as working memory, decision-making, problem solving, cognitive control, self-control and response inhibition [22–26]. Naturally, these cognitive functions, due to their complexity, depend on a large distributed brain network. Neuroimaging research showed that various frontal brain regions besides the DLPFC are linked to general cognitive control, including the anterior cingulate cortex [27], the ventrolateral prefrontal cortex [28], the orbitofronal cortex [29], the medial PFC [30] and the inferior frontal gyrus (IFG) [31]. These regions are highly functionally coupled with the DLPFC and most likely act in concert to sustain complex cognitive functions. In the context of eating behavior, neuroimaging evidence displays that DLPFC activity and functional coupling to the ventromedial PFC promote healthy food choices and successful dietary self-control [10, 25, 32]. DLPFC together with IFG activity are vital for the successful suppression of food craving and the motivation to eat [33–36]. There is some evidence supporting a left–right dichotomy showing that the right PFC to be more involved in inhibitory control and the left PFC in decision making processes as self-control abilities [10] Moreover, the PFC is responsive to a meal, postprandial hormones and to the taste and sight of food [37–43].

Persons with obesity fail to recruit left DLPFC activity particularly in response to food images [44] and to a meal [37, 45]. Moreover, obese patients with binge-eating disorder (BED) show an attenuated activation of the DLPFC, primarily in the right hemisphere, in response to a food-related response-inhibition task [46]. On the behavioral level, lower inhibitory capacity is linked to a higher Body-Mass-Index (BMI) [47, 48] and higher palatable food consumption [49]. Hence, cognitive control and its underlying neurobiological regulations is a relevant target to improve dysregulated eating and metabolic health.

Indeed, increased activity in the DLPFC and higher inhibitory control is related to successful self-control of food consumption. For instance, higher activation of the right DLPFC in response to high-calorie food images correlated with a subsequent reduction in *ad libitum* energy intake [50]. Shifting an individual’s attention to healthy eating increased left DLPFC activity [32, 51] and the capacity of activation in the DLPFC seems to be a predictor for weight loss success [52]. In this context, Weygandt et al. [53, 54] reported in two fMRI studies that higher activity in the DLPFC during a food-related decision making task is associated with the success of weight-loss and weight maintenance. Furthermore, adults with higher activation in the DLPFC while resisting food craving displayed better weight loss success following bariatric surgery [55]. In accordance, greater postprandial activation in the left DLPFC was reported in lean and post-obese women who successfully lost weight compared to obese women [45]. Besides being a significant predictor for weight loss success, it has been shown that it is possible to increase DLPFC activity using neurofeedback. A single training session was sufficient for overweight and obese adults to up-regulate their left DLPFC activity [56] as well as functional connectivity to the ventromedial PFC [57].

Taken together, the results suggest that the DLPFC and its functional connections to other frontal regions are vital for successful dietary self-control making this frontal network a prime target for the treatment of obesity. However, to date, it is not clear whether the right or left DLPFC contributes more significantly to dysregulated eating behavior and current literature hold evidence for both [10]. Even though, it is still not clear if failure to appropriately activate the DLPFC is a cause or consequence of obesity, overall, neuroimaging and behavioral data suggest that an increase in the activity of the DLPFC might be effective to lose and maintain body weight.

## Transcranial direct current stimulation (tDCS)

**Fig. 1:**
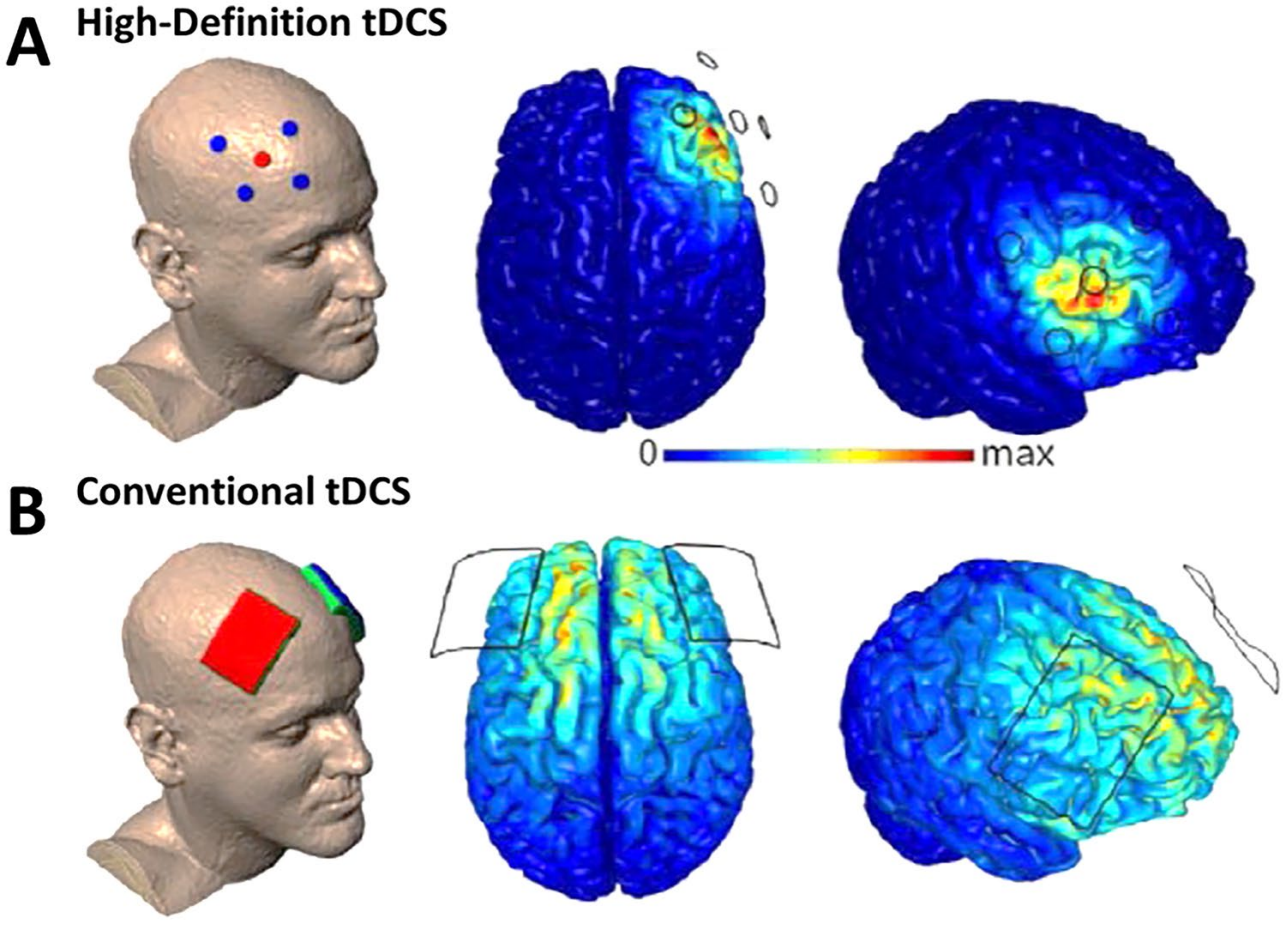
Finite element models of transcranial direct current stimulation (tDCS) montages aimed at targeting the dorsolateral prefrontal cortex (DLPFC). (**A**) The high-definition (HD) tDCS montages is displayed as a 4 × 1 ring montage. (**B**) The conventional tDCS montage shows two 5 × 7 cm sponge electrodes used in traditional tDCS. Electrode positions are based on the 10–20 international system. Conventional tDCS produces a wide-spread electric-field distribution compared to HD-tDCS which shows a higher focality of the target stimulation [65–67]. Figure is adapted from [86]

A growing number of studies in recent years have taken the approach of directly manipulating DLPFC activity using NIBS techniques such as repetitive transcranial magnetic stimulation (rTMS) and tDCS. Both approaches are able to modify cortical excitability in the brain [14–20]. Unlike rTMS, tDCS is less costly and easier to use [58–61] but cannot trigger an action potential [17]. The technique of tDCS relies on the application of a weak and constant direct current (DC) of mostly 1–2 mA for a duration of approximately 20 min, producing a weak electric field [14, 62, 63]. Usually conventional (i.e. traditional) tDCS deliver DC from a device using two large sponge electrodes [64]. However, a more recent tDCS design called high-definition (HD) uses multiple smaller electrodes [64], increasing focality compared to conventional tDCS [65–67]. The electrode formation mostly applied for HD-tDCS is the so-called 4 × 1 ring-configuration. The active electrode is placed over the target area while the four return electrodes are placed around the target, building a ring around the inner electrode [66, 68]. Figure 1 displays these two common tDCS technologies.

The underlying principle of action in tDCS is based on a subthreshold modulation of neuronal membrane potentials, leading to an alteration of the cortical excitability [69]. Nitsche and Paulus [14] demonstrated that this effect is polarity dependent with surface anodal tDCS resulting in an increase whilst suface cathodal stimulation showing a decrease of cortical excitability, also referred to as excitatory and inhibitory stimulation, respectively. However, this simple dichotomy of anodal-excitation and cathodal-inhibition (AeCi) is not apparent in all tDCS studies, and especially cathodal stimulation of higher intensities can produce excitatory effects; Batsikadze et al. [70] showed that the application of a 20-min cathodal tDCS, targeting the left primary motor cortex (M1), resulted in an enhancement of cortical excitability when using 2 mA current, while lower electric current (1 mA) decreased corticospinal excitability under cathodal stimulation.

Initial studies showed that the excitability-modulating effects of anodal tDCS outlasts the stimulation for up to 90 min [14, 63]. In addition to the acute effects, meaning the ability to modify excitability of the neurons, tDCS displays after-effects such as long term potentiation (LTP)-like plasticity in the human motor cortex, which can last for more than 24 h after stimulation [71, 72]. Accordingly, a review on physiological mechanisms of tDCS concluded that the effects are multifactorial and associated with GABAergic, serotonergic, glutamatergic, dopaminergic and cholinergic activity modulation [73]. For instance, Nitsche et al. [74] and Liebetanz et al. [75] showed that the after-effects of anodal and cathodal tDCS are influenced by an enhanced efficacy of the N-methyl-D-aspartate (NMDA) receptor, a member of the ionotropic glutamate receptors. This is of interest as NMDA receptors were shown to be involved in neuroplastic changes [76]. Moreover, recently published studies could show that the α-amino-3-hydroxy-5-methyl-4-isoxazolepropionicacid receptor (AMPAR), a crucial protein for enhancing synaptic transmission, is associated with tDCS induced plasticity in rodents [77, 78]. Taken together, these findings suggest that tDCS-related effects are not based on a single mechanism and involve a cascade of events at a molecular as well as on cellular level [73]. Generally, various parameters such as the current intensity, stimulation length and the number of sessions influence the duration of tDCS after-effects. However, the link between stimulation duration, current intensity and induced after-effects are more complex and increasing current strength [70, 79] or the stimulation duration [71, 80] do not necessarily show greater effectiveness. For instance, a study demonstrated that tDCS excitability effects are not linearly correlated with increasing current intensity [81].

Overall, conventional tDCS is known to be well-tolerated and with an applied current of 1–2 mA and a duration up to 20 min considered safe [82, 83]. Regarding HD-tDCS approaches, a study from Turski and colleagues [84] showed that 20 daily sessions of HD-tDCS in healthy adults administered over a variety of brain regions are safe and well tolerated. Concerning adverse effects (AE), a review concluded that most AEs are described as mild and short-lasting after stimulation [85].

### Methods

#### Search strategy

**Fig. 2:**
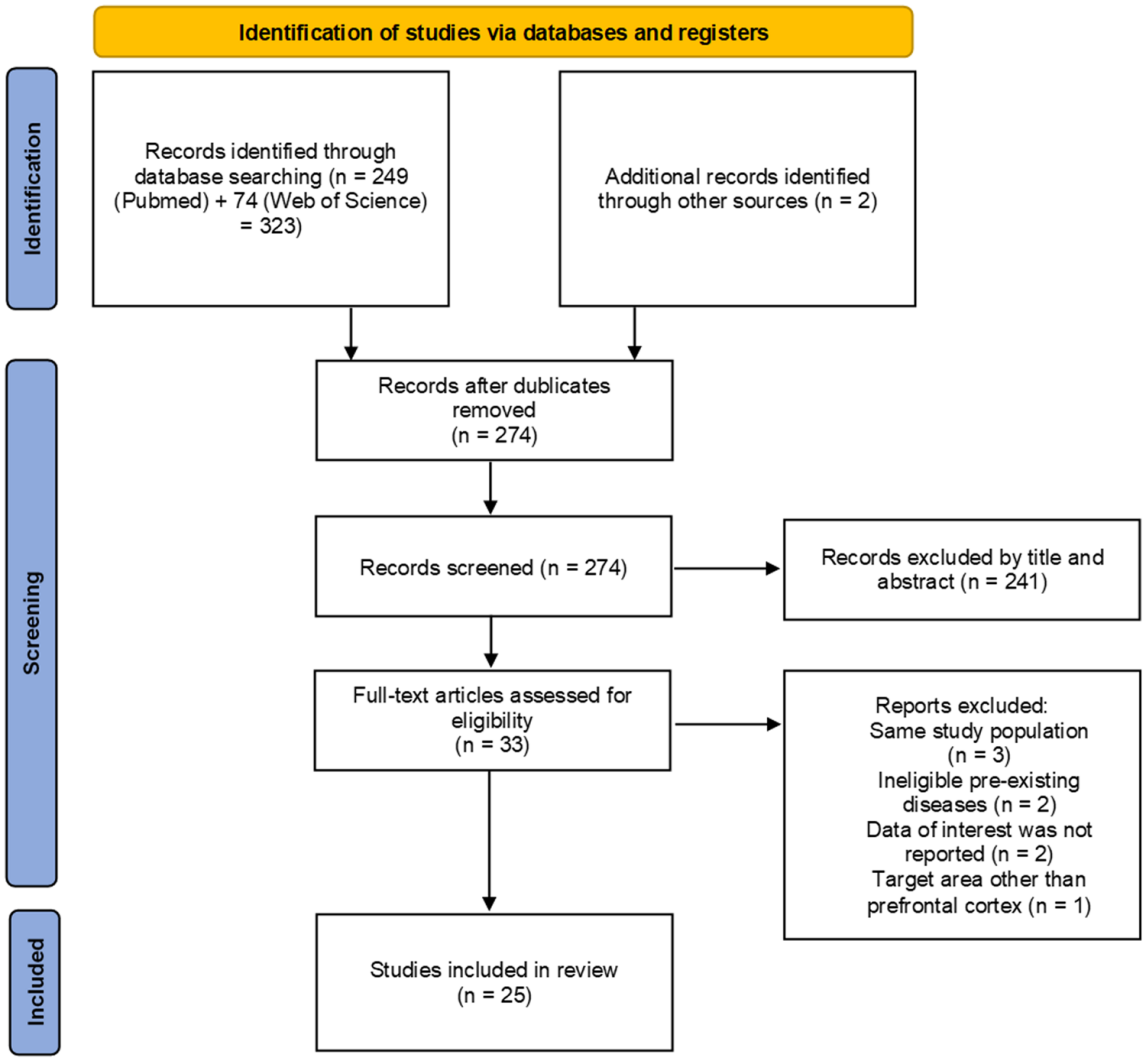
Flow chart of reviewed studies. Depicted are the reasons for exclusion and the final number of the included studies

This narrative review focused on the effects of tDCS aimed at the prefrontal cortex to influence food intake, food craving and body weight in healthy persons of different BMI groups. Only studies with tDCS as NIBS intervention were included in order to increase comparability of the already heterogeneous study designs. We sought to answer which tDCS protocols were able to influence eating behavior-related outcomes and show the most promising effects on weight-loss. In addition, this work elaborates possible modulators and limitations that influence tDCS effects with respect to eating behavior. To identify relevant studies, a two-staged literature search was carried out. First, an online search was conducted using the online databases Pubmed and Web of Science to cover articles published up to August 2021 with no starting date. For the PubMed search, the following search terms were used: "Transcranial Direct Current Stimulation"[Mesh] OR "transcranial direct current stimulation"[tw] OR "tDCS"[tw] OR "non invasive brain stimulation"[tw] OR "non-invasive brain stimulation"[tw] OR "NIBS"[tw] OR "brain stimulation"[tw] OR "neurostimulation"[tw] OR "neuromodulation"[tw] AND "Energy Intake"[Mesh] OR "Appetite"[Mesh] OR "eating behavior"[tw] OR "energy intake"[tw] OR "calorie consumption*"[tw] OR "caloric intake"[tw] OR "food addiction"[tw] OR "weight loss"[tw] OR "food craving*"[tw] OR "binge eating disorder"[tw] OR "food consumption"[tw] OR "appetite"[tw]. For the Web of Science search, the following search terms were used: *(((((((TI* = *(transcranial direct current stimulation)) OR TI* = *(tDCS)) OR TI* = *(non invasive brain stimulation)) OR TI* = *(non-invasive brain stimulation)) OR TI* = *(neuromodulation)) OR TI* = *(neurostimulation)) OR TI* = *(brain stimulation)) OR TI* = *(NIBS) AND (((((((((TI* = *(eating behavior)) OR TI* = *(energy intake)) OR TI* = (calorie consumption*)) OR TI = *(caloric intake)) OR TI* = *(food addiction)) OR TI* = *(weight loss)) OR TI* = *(food craving)) OR TI* = *(binge-eating disorder)) OR TI* = *(food consumption)) OR TI* = *(appetite).* Furthermore, review articles and meta-analysis were examined to identify additional articles. The identified studies were subsequently hand screened by reading the title and abstract and included in the study if they matched the research topic of our review. The remaining articles were evaluated in detail. A graphical depiction of the inclusion and exclusion process is illustrated in Fig. 2.

#### Selection criteria

In this review, all studies were included that met the following criteria: 1) randomized controlled trials and controlled clinical trials 2) tDCS was used as form of a non-invasive brain stimulation 3) food intake, hunger or appetite/desire to eat ratings, food craving changes or changes in body weight were measured 4) studies were placebo-controlled (i.e. sham stimulation as control condition). We excluded studies based on following criteria: 1) meta-analyses, reviews, case studies or meeting abstracts, 2) if participants were diagnosed with psychiatric disorders except for binge-eating disorder, 3) stimulation target other than the prefrontal cortex 4) animal studies, 5) studies not written in English.

#### Data extraction

Extracted data included the most important stimulation parameters: number of tDCS sessions, stimulation site (anode and cathode placement), current intensity, electrode size, duration of each session (min), total stimulation duration (min), tDCS montage (e.g. unilateral, bilateral), tDCS-Form (e.g. bipolar, HD-tDCS), between or within study-design and multi-session or single-session approach. Moreover, participant characteristics were included: population characteristics, number of subjects, and number of male and female participants, BMI and age. Lastly, factors related to the outcome measures were included (fasting time prior appointment) as well as the most important study results for the research question (weight loss, craving measurements, food intake measurements, desire to eat/hunger).

The literature search identified 25 studies that met the inclusion criteria. Most studies (*n* = 23) targeted the DLPFC (*n* = 7 targeted the left DLPFC, *n* = 15 targeted the right DLPFC, *n* = 1 targeted both, left and right DLPFC), while two studies targeted the right IFG.

## tDCS effects on food craving and desire to eat

Food craving and food cue reactivity have shown to predict caloric intake and weight gain [87]. Persons with elevated BMI and BED display higher food craving [88–90]. Even though the theory that foods can trigger an addictive process remains controversial, neurobiological evidence shows similar neural pathways between food craving and drug craving [12, 91, 92]. These include regions implicated in homeostasis, motivation, reward and emotions, which are deeply located in the subcortical part of the brain and are therefore difficult to reach with conventional tDCS methods. Nonetheless, cortical regions, such as the frontal cortex, can be easily targeted with tDCS and are functionally connected to other regions in the brain [14, 63, 93, 94]. It is hypothesized that targeting frontal brain areas improves cognitive control and response inhibition [95–97]. Consistent with this, a recent meta-analysis confirmed that a single tDCS application has a significant overall effect on inhibitory control [98].

**Fig. 3:**
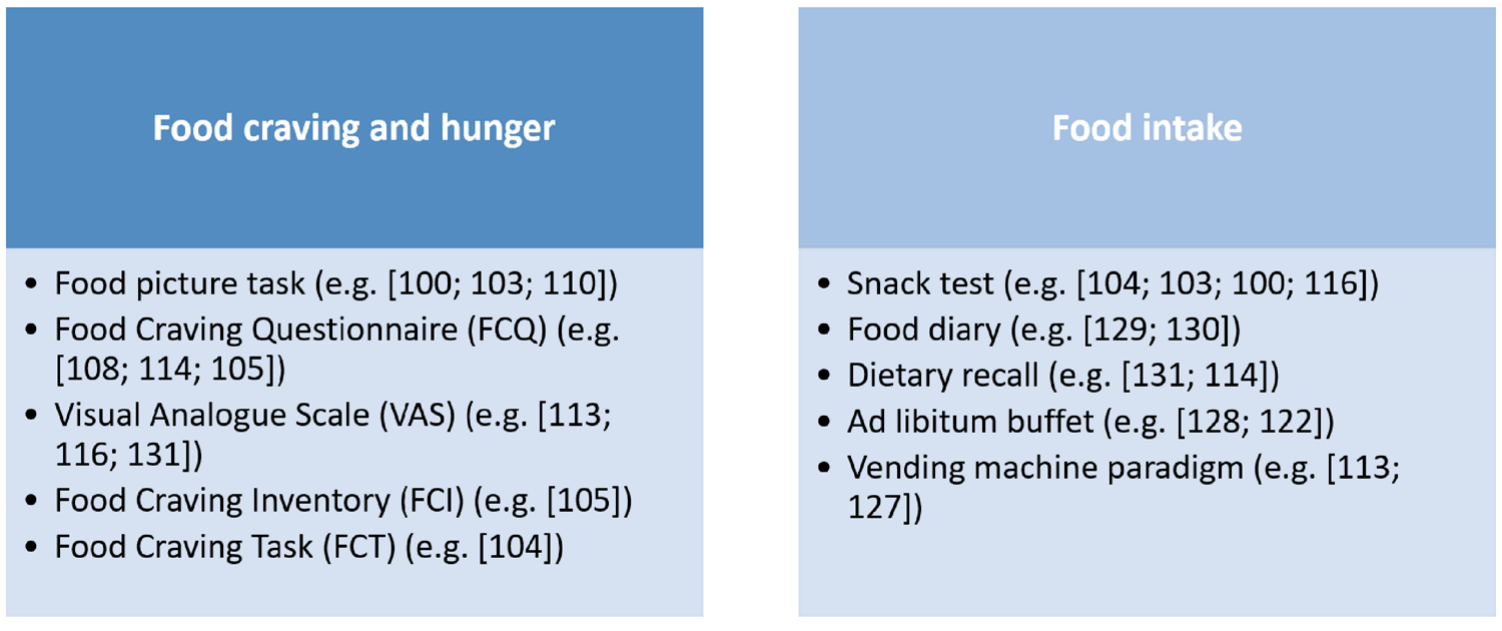
Overview of tools used to evaluate eating behavior in response to tDCS

Food craving can be measured using questionnaires such as the Food Cravings Questionnaire–State (FCQ-S) or by using a visual analogue scale (VAS). Other craving measurements include visual food reactivity tasks where images display different kinds of food (e.g. savory foods, dessert and non-sweet carbohydrates) on a screen and participants are asked to rate on a VAS to what degree they “like” and “want” the presented food before and immediately after stimulation. For evaluation, the “wanting” scores are used, as these are considered to reflect food craving. Figure 3 provides an overview of the tools used to assess eating behavior in response to tDCS. The majority of tDCS studies examining the effects on food craving targeted the right DLPFC [99–110], as it is associated with inhibitory control and reward-based learning [10, 111]. Some trials aimed to enhance self-control by stimulating the left DLPFC [112–114, 116, 157], or targeting both sites [116] by using anodal stimulation at the right and left DLPFC, respectively.

One study, however, stimulated specifically the right IFG [114], which is involved in response inhibition [117]. Recent findings show that anodal tDCS over the IFG can facilitate response inhibition by modulating neural activity and functional connectivity to other brain regions [118]. However, anodal stimulation of the right IFG had no effect on food craving [114]. See Table 1 for an overview of the study population and design and Supplementary Table 1, which provides an overview of the targeted brain areas and stimulation parameters. Table 2 presents the individual study findings summarized in this review.

**Table 1:** Overview study population and design

**Author**	***N* Intervention/ *N*** control	**Study design**	**Sex F/M**	**Population**	**Mean Age** **Intervention/control**	**Mean BMI** **Intervention/control**	**Single/** **Multi-** **sesssion**	**Fasting before tDCS**
**tDCS only**								
Burgess et al. 2016 [99]	30/30	within	20/10	Overweight subjects with BED/subBED	adults	36.1 ± 6.12^a^	S	Eat 3 h prior visit
Beaumont et al. 2020 [100]	21/21	within	11/10	Healthy, normal-weight subjects not prone to overconsumption	24 ± 7^a^	22.8 ± 2.3^a^	S	Min. 4 h
Chen et al. 2019 [114]	28/29	between	57/0	Restrained eaters	20.96 ± 1.86/20.52 ± 1.55^a^	21.75 ± 2.34/21.46 ± 2.61^a^	S	Min. 3 h
Fregni et al. 2008 [116]	23	within	21/2	Healthy subjects	23.7 ± 7.2^a^	Not specified	S	3 h
Forcano et al. 2020 [128]	9/9	between	12/6	Morbid obese subjects undergoing bariatric surgery	43.7 ± 9.0/43.2 ± 10.6^a^	43.17 ± 5.7/41.94 ± 4.0^a^	M	Eat 2-4 h prior visit
Georgii et al. 2017 [101]	42/42	within	42/0	Predominantly healthy women (underweight, normal weight, overweight and obese subjects)	22.02 ± 4.25^a^	22.6 ± 3.09^a^	S	3 h
Gluck et al. 2015 [126]	5/4	between (+ within)	6/3	Healthy, obese subjects	42 ± 8^a^	38 ± 7^a^	M	Overnight fast
Goldman et al. 2011 [102]	19/19	within	13/6	Healthy subjects with frequent food craving	32.47 ± 10.85^a^	27.25 ± 6.24^a^	S	4 h
Grundeis et al. 2017 [127]	25/25	within	25/0	Obese women	28.8 ± 6^a^	36.5 ± 4.1^a^	S	Min. 5 h
Heinitz et al. 2017 [112]	29	between	17/12	Obese subjects	35.55 ± 9.07^a^	38.9 ± 6.68^a^	M	Overnight fast
Jauch-Chara et al. 2014 [121]	14/14	within	0/14	Healthy normal-weight men	24.81 ± 0.58^b^	22.65 ± 0.34^b^	M	6 h
Kekic et al. 2014 [103]	17/17	within	17/0	Healthy women with frequent food craving	26.41 ± 8.31^a^	23.81 ± 2.60^a^	S	Not specified
Lapenta et al. 2014 [110]	9/9	within	9/0	Healthy normal-weight women with frequent food craving	23.4 ± 2^a^	21.9 ± 1.63^a^	S	3 h
Ljubisavljevic et al. 2016 [104]	13/14	between	8/19	Normal weight and overweight subjects with frequent food craving	21 ± 2.1/21.6 ± 2^a^	26.3 ± 5.1/24.9 ± 3.6^a^	M	Eat 3-4 h prior visit
Marron et al. 2019 [158]	12/12	within	9/3	Obese subjects	41.6 ± 4.8^a^	32.7 ± 1.9^a^	S	4 h
Ray et al. 2017 [106]	18/18	within	10/8	Obese subjects with frank obesity	22.7 ± 7.9^a^	37.4 ± 9.1^a^	S	Neither hungry nor full
Ray et al. 2019 [105]	39/35	between	44/30	Overweight and obese subjects	19.9 ± 3.4^a^	31.8 ± 5.5^a^	S	Neighter hungry nor full
Sedgmond et al. 2019 [108]	88/84	between	134/38	Healthy subjects	20.51 ± 0.28/21.1 ± 0.45^b^	23.24 ± 0.41/22.52 ± 0.38^b^	S	3 h
Sedgmond et al. 2020 [107]	55/55	within	42/13	Underweight, normal weight, overweight and obese subjects	22.25 ± 0.76^b^	23.34 ± 0.43^b^	S	3 h
Stevens et al. 2020 [109]	28/28	within	19/9	Overweight and obese subjects	21	34 ± 7.05^a^	S	Neither hungry nor full
To et al. 2018 [125]	23	within	23/0	Healhty women; Restrained eaters	24.7 ± 4.2^a^	24.3 ± 4.3^a^	S	Not specified
**Combination tDCS + hypocaloric diet**								
de Araujo et al. 2020 [129]	14/14	between	14/14	Overweight and obese subjects	37.5 ± 7/37.7 ± 4.7^a^	31.8 ± 2.6/31.3 ± 2.4^a^	M	3 h
Fassini et al. 2020 [113]	20/18	between	38/0	Healthy obese women	32.16 ± 1.17/30.61 ± 1.21^b^	33.27 ± 0.30/32.98 ± 0.21^b^	M	2 h
Amo Usanos et al. 2020 [115]	20/18	between	38/0	Healthy overweight and obese women	53.1 ± 1.3/51.8 ± 1.3^b^	31.5 ± 0.6/31.9 ± 0.7^b^	M	Not specified
**Combination tDCS + exercise**								
Montenegro et al. 2012 [130]	9/9	within	4/5	Overweight subjects	28.2	24	S	2-3 h

*BED* Binge Eating Disorder, *BMI* Body Mass Index, *F* Female, *M* Male, *M* Multisession approach, *S* Single-session approach, *tDCS* transcranial Direct Current Stimulation

^a^Values are expressed as means ± SD

^b^Values are expressed as means ± SEM

**Table 2:** Main outcomes active tDCS vs. sham

**Autho**r	**Main findings weight loss**	**Measures food craving**	**Main findings food craving**	**Measures food intake**	**Main findings food intake**	**Measures desire to eat/hunger**	**Main findings desire to eat/hunger**
**Stimulation type**							
Burgess et al. 2016 [99] Anodal right DLPFC	Not measured	Food picture task	- reduced food craving ratings - reduced food craving ratings for dessert, savory protein and all-foods but not for carbohydrates - craving ratings in men were more reduced compared to women for dessert and all-foods	Snack test	- reduced kcal intake by 11% - no reduction in a particular food-type - reduced kcal intake of preferred food	At-Home Binge-Eating Survey (incl. desire to binge-eat)	- reduced desire to binge-eat only in men
Beaumont et al. 2020 [100] Anodal right DLPFC	Not measured	FCQ-S	- no effects of pre- vs. post ratings (not compared to sham)	Not measured		Hunger (VAS) Desire to eat (VAS)	- no effects of pre vs. post ratings (not compared to sham)
Chen et al. 2019 [114] Anodal right IFG	Not measured	FCQ-S	- no main effects - overall increase in FCQ-S scores from pre- to post measurements (real and sham tDCS)	Not measured		Hunger (VAS) Desire to eat (VAS)	- no effects on hunger and desire to eat - overall increase in desire to eat palatable foods from pre to post measurements (real and sham tDCS)
Fregni et al. 2008 [116] Anodal right and left DLPFC	Not measured	VAS	- reduced craving ratings only after anode right/cathode left	Snack test	- reduced kcal intake	Not measured	
Forcano et al. 2020 [128] Anodal right DLPFC	- no effects for active vs. sham	Not measured		Food diary	- reduced post intervention kcal intake - further reduced kcal intake during follow-up - mostly due to a reduced fat intake - higher sugar intake post intervention	Not measured	
Georgii et al. 2017 [101] Anodal right DLPFC	Not measured	FCQ-S (short version)	- no effects	Snack test	- no effects	Desire to eat (VAS)	- no effects
Gluck et al. 2015 [126] Anodal and cathodal left DLPFC	- no effects for active vs. sham - more weight loss after anodal compared to cathodal tDCS	Not measured		Vending machine	- no effects for active vs. sham - reduced kcal intake from fat and soda during anodal compared to cathodal tDCS	Not measured	
Goldman et al. 2011 [102] Anodal right DLPFC	NM	Food picture task	- reduction in pre- to post food craving - reduction in pre- to post food craving for sweet food and carbohydrates but not for fast-food and high-fat food images	Snack test	- no effects	Not measured	
Grundeis et al. 2017 [127] Anodal and cathodal left DLPFC	Not measured	Not measured		Buffet	- no effects - no effects on specific food groups (high caloric sweet/salty and low caloric sweet/salty)	Hunger (VAS)	- no effects
Heinitz et al. 2017 [112] Anodal left DLPFC	- no effects during inpatient stay and after 4-week outpatient stay	VAS	- no effects after 3 tDCS sessions and after 15 tDCS sessions	Vending machine and Snack test	- no effects on *ad libitum* food intake from a vending machine during 3 consecutive days of tDCS - no effects on a snack test after 3 consecutive days of tDCS - fewer kcal intake during a snack test after 15 sessions of tDCS - less kcal intake from candy during a snack test after 15 sessions of tDCS	Hunger (VAS) Urge to eat (VAS)	- no effects on hunger and urge to eat ratings after 3 tDCS sessions - hunger and urge to eat ratings decreased significantly more during the study in the active group
Jauch-Chara et al. 2014 [121] Anodal right DLPFC	- no effects	Not measured		Buffet	- no effects after 1 tDCS session - less total kcal intake after 8 days of consecutive stimulation - reduced kcal intake mostly due to a reduced intake in carbohydrates	Hunger (VAS) Appetite (VAS)	- no effects on hunger after 1 and after 8 tDCS sessions - no effects on appetite scores after 1 tDCS session - reduced non-specific appetite scores after 8 sessions of tDCS - reduced appetite scores for sweet and savory food after 8 sessions of tDCS
Kekic et al. 2014 [103] Anodal right DLPFC	Not measured	FCQ-S and FCT	- reduced FCQ-S scores by sham (mostly due to FCQ-S subscale 5) - no effects for global FCT-scores - lower FCT scores for sweet food - reduced FCT scores for savory food in real and sham condition (pre vs. post stimulation)	Snack test	- no effects - no effects on kcal intake from specific food groups (crisps, chocolate, nuts, biscuits) between conditions	Hunger (VAS)	Only measured before stimulation
Lapenta et al. 2014 [110] Anodal right DLPFC	Not measured	Urge to eat (VAS)	- reduced urge to eat ratings	Snack test	- reduced total kcal intake	Not measured	
Ljubisavljevic et al. 2016 [104] Anodal right DLPFC	Not measured	FCQ-S, FCQ-T, FCI	- FCQ-S: reduced food craving scores after a single session of active tDCS (pre vs. post stimulation) - FCQ-T: reduced craving ratings after 5 days of consecutive active tDCS compared to baseline - FCQ-S and FCQ-T: reduced craving ratings at follow-up - FCI: reduced craving for sweet, fast food and fat but not carbohydrates	Not measured		Not measured	
Marron et al. 2019 [158] Anodal left DLPFC	Not measured	Not measured		Not measured		Appetite (VAS)	- increase in appetite - increase in cue-triggered hunger in pre vs. post ratings (not compared to sham) - increase in cue-triggered desire to eat in pre vs. post ratings (not compared to sham)
Ray et al. 2017 [106] Anodal right DLPFC	Not measured	Food picture task	- no main effects - reduced food craving in women with reduced attention-type impulsiveness	Snack test	- no main effects - reduced food intake for preferred foods in men with lower intention to restrict kcal intake - reduced total food intake in men with higher non-planning-type impulsiveness	Hnger (VAS)	Only measured before stimulation
Ray et al. 2019 [105] Anodal right DLPFC	Not measured	Food picture task	- no effects - effect of expectation on craving for all food types – less craving in subjects who were told to be stimulated with real tDCS	Snack test	- no overall effects - effect of expectation on kcal intake - less total kcal intake in subjects who were told to be stimulated with real tDCS	Not measured	
Sedgmond et al. 2019 [108] Anodal right DLPFC	Not measured	FCQ-S	- no effects	Snack test	- no main effects - more kcal consumption of healthy food - no effect for real vs. sham tDCS on food type (sweet vs. savory food)	Hunger (VAS) Desire to eat (VAS)	- no effects
Sedgmond et al. 2020 [107] Anodal right DLPFC	Not measured	FCQ-S	- no effects	Not measured		Desire to eat (VAS)	- no main effect on desire to eat ratings for real vs. sham tDCS - no effect on desire to eat sweet/savory food for real vs. sham tDCS
Stevens et al. 2020 [109] Anodal right DLPFC	Not measured	Food picture task	- no effects - no effect for real vs. sham tDCS on food type (sweet, fatty protein, carbohydrates, mixed macronutrients)	Snack test	- no effects	Hunger (VAS)	Only measured before stimulation
To et al. [125] Anodal right IFG	Not measured	FCQ-S	Only measured before stimulation	Chocolate snack test	- increase in chocolate consumption	Hunger (VAS)	Only measured before stimulation
**Combination tDCS + hypocaloric diet**							
de Araujo et al. 2020 [129] Anodal right DLPFC	- no effect (higher reduction in body weight of the active group was not significant)	Not measured		Food diary	- no effect in total energy intake throughout the study - no effect in macronutrient intake throughout the study	Hunger (VAS) Desire to eat (VAS)	- no effect on hunger ratings throughout the study - reduced desire to sweet food after 4 weeks of tDCS - no effects on desire to eat savory, salty or fatty foods at the end of the study
Fassini et al. 2020 [113] Anodal left DLPFC	- no effect during intervention - effect on weight loss at 6-month follow-up where the sham group lost more weight compared to the active tDCS group - more participants stimulated with real tDCS regained weight (measured at 6-month follow-up)	FCQ-S	- no effects	Dietary recall	- no effects	Hunger (VAS) Desire to eat (VAS)	- no effects
Amo Usanos et al. 2020 [115] Anodal left DLPFC	- reduction in body weight throughout the study	FCQ-S	- no effects	Not measured		Hunger (VAS) Desire to eat (VAS)	- no effects after one and two weeks of tDCS - trend towards lower desire to eat ratings after 8 sessions
**Combination tDCS + exercise**							
Montenegro et al. 2012 [130] Anodal left DLPFC	Not measured	Not measured		Dietary recall	- no findings mentioned	Hunger (VAS) Desire to eat (VAS)	- tDCS with subsequent exercise decreased hunger directly after aerobic exercise - tDCS with subsequent exercise decreased desire to eat more than tDCS or exercise alone

*FCI* Food Craving Inventory, *FCT* Food Challenge Task, *FCQ-S* Food Craving Questionnaire – State, *FCQ-T* Food Craving Questionnaire – Trait, *tDCS* transcranial Direct Current Stimulation, *VAS* Visual analogue scale

Overall, the main findings of tDCS on food craving show mixed effects, varying from significant reductions in food craving by active tDCS [99, 102, 104, 110, 116] to null findings [100, 101, 103, 105, 107–109, 113, 114, 116] or showing only significance for a specific group of individuals [106]. Even an opposite effect, favoring sham over anodal tDCS of the right DLPFC was shown in a single-session study [103]. However, these findings were mostly based on a subscale “craving as a physiological state” in the FCQ-S questionnaire, which contains statements about objectively determinable hunger, such as feeling weak as a result of food deprivation. When excluding this subscale from the analysis, the differences between sham and active tDCS were no longer significant. Moreover, the study used next to the FCQ-S a modified Food Challenge Task (FCT) to measure food craving which did not reveal differences between sham and active tDCS [103].

In a clinical trial from Burgess et al. [99], food craving and the desire to binge-eat was reduced after a single stimulation of anodal tDCS compared to sham targeting the right DLPFC. This effect was sex-specific, showing that men were more responsive compared to women regarding tDCS effects on desire to binge-eat and food craving [99]. Sex-effects were also reported by Ray et al. [106], where a reduction in food craving was only displayed in women with low attentional impulsivity. Other studies have also demonstrated sex-specific effects of tDCS on different cognitive domains. For instance, one study revealed that men benefit from tDCS stimulation of the left DLPFC, whereas women profit from right DLPFC stimulation in terms of verbal working memory [119]. Among the various explanations discussed for the differential effects of tDCS in men and women is the influence of sex hormones and neurotransmitters [120].

Despite the heterogeneous results of tDCS on food craving [100, 101, 103, 105, 107–109, 113, 114, 116], some studies identified an effect of active tDCS on food craving and appetite on specific foods [99, 102–104, 121]. In particular, the reduction in cravings for sweet foods such as desserts appear to be fairly consistent and has been observed in several tDCS studies aimed at the right DLPFC. Therby, it does not seem to matter whether the subjects are obese or not, as the effects were demonstrated in both subjects of normal-weight and obesity. Most studies showing this effect examined participants experiencing frequent cravings, BED, or sub-BED [99, 102–104]. Nevertheless, a study with healthy normal-weight men also showed decreased appetite for sweets after 8 consecutive anodal tDCS sessions [121]. Contrary, a study including overweight and obese subjects without eating disorder could not demonstrate this effect on sweet food after anodal tDCS [109]. For other food categories such as savory food, the data are not as consistent [102, 104]. However, cravings for chocolate and sweets have been shown to be more prevalent compared to craving for savory foods [122] which may explain the mixed results for sweet foods compared to other food categories.

Taken together, the current literature of tDCS studies targeting the left or right DLPFC show conflicting results with respect to food craving. One possible explanation for this variability could be the highly diverse study population. For instance, studies showing a diminished craving for sweet foods included mostly participants with either frequent food craving [102–104], BED or subBED [99], thus demonstrating a study population that exhibits dysregulated eating behaviors. A recently published meta-analysis evaluated the effects of modulators in tDCS studies on food and substance craving and concluded that the stimulation site (anodal left or right DLPFC) and current intensity (1 or 2 mA) do not influence tDCS outcomes [123]. However, stimulation duration made a significant difference, meaning that a longer total stimulation time was associated with a stronger craving reduction [123]. In addition, studies with multiple sessions showed a better effect on craving than stimulations with only one session. [123, 124].

## tDCS modulating food intake

Much effort is being made to evaluate tDCS protocols on food intake measures in order to establish new treatment strategies for persons with obesity and eating disorders. So far, most studies evaluated anodal tDCS over the right DLPFC (for overview see Supplementary Table 1). For this purpose, studies use mostly snack tests [99, 101–103, 105, 106, 108–110, 112, 116, 125], vending machine paradigms [112, 126], *ad libitum* test buffets [121, 127], dietary records or dietary recalls [113, 128–130] (see Fig. 3, Table 2).

### Snack test

For in-lab food consumption, snack tests are commonly used. Participants are usually left alone in a test room for a certain amount of time (~ between 10 and 20 min) to consume the served snacks *ad libitum.* The consumed calories are calculated afterwards. In some studies, the true reason for the snack test was masked. Subjects are then asked to rate the snack for taste and palatability [99, 106, 109, 112, 125], which is a valid measure of food consumption [131].

Active tDCS aimed at the right DLPFC reduced craving ratings and appetite for highly palatable food [99, 102–104, 121]. Actual food consumption (i.e. chocolate), on the other hand, was increased after active tDCS compared to sham in participants who reported to frequently crave chocolate [125]. However, in this study, tDCS was directed at the right IFG, whereas the aforementioned studies investigated food craving in response to tDCS covering the right DLPFC, making a direct comparison of these study results difficult.

A multi-session study showed after four weeks of anodal tDCS over the left DLPFC (15 sessions in total), that obese participants consumed less snacks compared to subjects of the sham group, particularly less sweet foods such as candy [112]. Interestingly, no significant lower consumption of snacks was observed after only three consecutive tDCS sessions between the sham and active tDCS group [112]. This may indicate that the effects of tDCS become apparent only after a certain number of sessions. In fact, a recent meta-analysis confirmed that repeated tDCS and rTMS sessions have a stronger effect on food consumption than single-session studies [124]. Nevertheless, studies using single-session tDCS protocols seem to be effective as well [99, 110, 116]. However, a large study including 172 participants in the analysis failed to show a reduced snack food intake after a single application of anodal tDCS targeting the right DLPFC [108]. Other studies also failed to demonstrate reduced snack intake after anodal tDCS in single-session approaches [101, 102, 106]. Furthermore, the expectation of receiving active stimulation seemed to influence the actual food intake. Informing participants that they will receive active tDCS resulted in decreased food intake, regardless of whether subjects actually received real or sham tDCS [105].

### Vending machine paradigm

Vending machine paradigms can be used to accurately measure food intake throughout the day [132, 133]. In this approach, each participant has *ad libitum* access to a vending machine for a specified period of time (e.g. 23.5 h after completion of the tDCS session). The vending machine is filled with a variety of foods previously selected based on personal ratings in a Food Preference Questionnaire [134], as well as additional beverages. For food intake, subjects are asked to consume all meals in a specific room and are not allowed to use electronic devices. Moreover, participants have to return any food not consumed in order to evaluate total caloric intake.

Two studies by the same working group used a vending machine paradigm to assess complete caloric intake during three days of consecutive tDCS in obese participants [112, 126]. Thereby, subjects were in an inpatient setting for nine [126] and 11 days [112] and received a weight-maintaining diet for the first five [126] and seven [112] days. Subsequently, active anodal tDCS and cathodal tDCS, respectively, or sham stimulations targeting the left DLPFC were performed in the morning on three consecutive days. After the respective tDCS sessions, participants had the opportunity to eat *ad libitum* products of a vending machine. However, no effect was observed on caloric intake between active and sham stimulation [112, 126]. Gluck et al. [126] though, identified a trend towards lower total caloric intake in response to anodal compared to cathodal stimulation. In addition, the anodal tDCS group consumed significantly fewer calories from fat and soda compared to participants receiving cathodal tDCS [126]. When interpreting these data, the inpatient setting should taken into account. Robinson et al. [135] showed that increased awareness of being observed caused reductions in caloric consumption.

### Test buffet

In contrast to snack tests, a test buffet usually contains a larger selection of foods. Here, too, the participants have the opportunity to consume the foods presented *ad libitum* for a certain period of time, which may be longer as during a snack test.

To our knowledge, two studies assessed food intake in response to tDCS using a test buffet. Jauch-Chara et al. [121] showed that eight days of consecutive anodal tDCS targeted at the right DLPFC could reduce caloric intake in young, normal-weight men compared to eight days of sham tDCS in a crossover-design study. The reduced caloric intake was mostly due to a reduced intake of carbohydrates [121]. In another study including women with obesity, subjects received in a random order single anodal, cathodal, and sham tDCS aimed at the left DLPFC with subsequent ad libitum *ad libitum* buffet after stimulation. Results did not show differences in the overall caloric intake between conditions [127].

### Dietary record and dietary recall

Some intervention studies use self-reported dietary records to assess participants' dietary intake in their free-living environment. In this process, subjects typically weigh all the foods and beverages they consumed over a specified period of time. A dietary recall, on the other hand, usually consists of a guided interview by which participants list all foods and beverages consumed in the last 24 h. Several studies examined the effects of tDCS on food intake behaviors using these assessments.

For instance, one study aimed at enhancing the right DLPFC in morbid obese patients using a HD multichannel tDCS configuration to increase focality. Therby, patients received four days of consecutive tDCS combined with a cognitive training prior to bariatric surgery. Active stimulated patients reduced their caloric intake measured by a food diary compared to subjects who received sham. Interestingly, this effect was stronger at follow-up [128]. Another multi-session tDCS study administered daily anodal or sham tDCS targeting the right DLPFC over four weeks (20 sessions in total) in overweight and obese subjects. Next to tDCS as an intervention, participants had to follow a hypocaloric diet. No differences between stimulation conditions were observed on food intake based on a 3-day weighed dietary record [129]. Other studies failed to show an effect of anodal tDCS on food intake as well [113].

Together, these findings show that the effects of tDCS on caloric intake are highly variable. A meta-analytic review concluded that for single-session tDCS and rTMS approaches targeting the DLPFC no causal effect on food consumption could be confirmed [136]. In addition, a more recent meta-analysis from Song et al. [124] evaluated the effects of excitatory tDCS and rTMS aimed at the DLPFC on craving and consumption in persons suffering from eating disorder, obesity or drug addiction. There, a significant effect of non-invasive neurostimulation on consumption, including drug consumption such as alcohol and nicotine, was found for single-session studies as well as for multi-session approaches. Restricting the analysis to food intake revealed a significant reduction in consumption with a medium effect size [124].

## The impact of tDCS on body weight

The ultimate goal of evaluating novel tDCS paradigms is to achieve clinical relevant effects as successful weight loss or body weight maintenance. A number of studies assessed the impact of tDCS on body weight changes [112–114, 116, 121, 126, 129, 157]. Here, only few trials stimulated the right DLPFC [121, 129] while most studies conducted tDCS of the left DLPFC [112–114, 116, 126, 157].

In an inpatient-design study, three days of consecutive tDCS of the left DLPFC did not influence body weight in the active tDCS groups (anodal and cathodal) compared to subjects receiving sham stimulation [126]. Despite the lack of differences between active and sham tDCS, subgroup-analysis comparing anodal vs. cathodal stimulation revealed that obese subjects receiving anodal tDCS showed a significant higher weight loss [126]. One study published at a later time by the same research group evaluated only the effects of anodal vs. sham tDCS aimed at the left DLPFC and showed no differences in weight change after 15 sessions of stimulation in the anodal tDCS group compared to the sham group [112]. Consistent with these results, eight days of consecutive active anodal tDCS aimed at the right DLPFC had no impact on body weight compared to sham stimulation in a crossover-design study. However, the study was conducted in healthy normal-weight men [121]. Hence, no conclusion can be drawn whether persons with obesity could reduce their body weight with this tDCS protocol.

In addition to tDCS as a stand-alone intervention, there are also studies examining the effects of tDCS in combination with a hypocaloric diet on weight loss. A recent trial conducted from Amo Usanos et al. [115] involved overweight and obese women and investigated the effects of a 4-week trial with a total of eight tDCS sessions. Here, participants received in the first week five consecutive tDCS sessions and in the second week three tDCS sessions combined with a hypocaloric diet followed by two weeks of a hypocaloric diet only. Both groups reduced body weight but the active group showed a significant greater reduction than the sham group throughout the study [116]. Another recent trial examining tDCS effects in combination with a hypocaloric diet in overweight and obese participants could not confirm these findings after 20 sessions of active or sham tDCS. Although the active group lost more weight, the differences did not reach statistical significance [129]. However, it should be mentioned that the two studies are difficult to compare, as different sides of the DLPFC were anodal stimulated (right [129] vs. left [116]). Moreover, in the study by Amo Usanos et al. [115] all subjects were exclusively female, whereas in the study by de Araujo et al. [129] 50% of the subjects were male, thus increasing interindividual variability.

We are not aware of any meta-analysis that examined the effects of tDCS on weight loss. Nevertheless, existing literature suggest that there are several modulators that contribute to the results of each study. Future studies should focus on the modulators affecting the outcomes of tDCS studies regarding weight loss.

## Limitations

There are multiple factors and modulators which may account for the variability of the outcomes in studies investigating the effects of tDCS on eating behavior and weight-loss.

### Single vs. multi-session approaches

First, some studies investigated tDCS effects using a single-session tDCS design while other trials used multi-session approaches (see Table 1 for overview). Meta-analytic approaches already elucidated that multi-session design studies have stronger effects compared to single-session stimulations regarding the reduction of food craving and consumption [123, 124].

### Current strength

While most studies used 2 mA, some trials examinated tDCS effects on eating behavior using lower intensities [101, 107, 114, 121]. These studies did not report an effect of single session active tDCS vs. sham stimulation on food craving [101, 107, 114], food consumption [101, 121], desire to eat [101, 107, 114] or hunger [114, 121]. However, Jauch-Chara et al. [121] were able to detect significant effects on food intake and appetite scores after the completion of eight tDCS sessions using a DC of 1 mA. One possible explanation for null-findings after single-session tDCS could be an insufficient current strength. Before the current reaches the targeted brain region, the electric field distribution during tDCS is determined by several factors like the gyral depth, the thickness of the skull, and the cerebrospinal fluid as well as the distance from anode to cathode. This could be shown by using anatomically realistic finite element models (FEM) [137]. Moreover, other factors such as head fat were shown to affect tDCS electric current density across the brain [138]. In addition, a recently published study reported that only about 25% of the applied current reaches the brain [139] and Hall and Lowe [140] concluded that 1 mA is an insufficient current for affecting brain networks relevantly [139]. However, it is crucial to mention that this conclusion was drawn based on human post mortem brain tissue. Contrary to the prior conclusion, a study investigating the effects of different stimulation intensities (0.5 – 2.0 mA) for anodal and cathodal tDCS found that lower intensities (0.5 and 1 mA) displayed equal effects in the excitability of the motor cortex. Moreover the researchers showed that stimulation effects were not correlated with increased DC intensities [81].

### tDCS method and further directions

Non-invasive brain stimulation techniques such as tDCS can alter cortical activity through electrical current with the potential to induce long-lasting behavioral effects. A majority of the tDCS studies used conventional tDCS with two large sponge electrodes. However, this stimulation montage results in a diffuse brain current flow [66]. Novel technologies such as HD-tDCS overcome the diffuse electric -field by using multiple smaller electrodes, which have shown to increase focality [66, 68, 141] (see Fig. 1). First results are promising for multisession approaches [128].

In terms of eating behavior regulation and inhibitory control, the DLPFC represents an important target area for stimulation, and indeed applying tDCS to this brain region has been shown to reduce food craving and intake [124]. Nevertheless, it is well-known that the human brain is organized in functional networks rather than working in isolation [142–145] with the DLPFC being only one player in the regulation of human eating behavior [146]. Therefore, brain stimulation methods are needed that allow modulation of network activity as a whole rather than stimulating isolated brain regions (see [94] for review). Fischer et al. [147] could show that multifocal tDCS targeting the left M1 and its associated network more than doubled the increase of the excitability over time in the aimed brain area compared to traditional tDCS. Moreover, Dagan et al. [148] compared the effects of tDCS over M1 (single-target) to a multi-target stimulation of M1 and the DLPFC using HD-tDCS on cognitive and motor function in patients with Parkinson’s disease. Here, a single-session of multi-target stimulation of both brain areas showed a significant improvement of the outcomes compared to the single-target intervention. Overall, network-targeted tDCS is a promising method to enhance tDCS effects. Further investigations are needed to determine if these effects can be demonstrated for other brain networks and at the behavioral level.

### tDCS combined with different interventions

Another explanation of the heterogeneity of tDCS outcomes include methodological variability. Thereby it has been shown that the administration of a task, for instance go/nogo tasks, may play a role in the different outcomes between tDCS studies [149]. Moreover, some studies combine tDCS together with a hypocaloric diet or exercise. A recent review concluded that a combination of tDCS and aerobic exercise may have beneficial synergistic effects on cognition [150]. However, it is not clear if this is also the case for eating behavior related tDCS outcomes.

### Stimulation site of tDCS

Moreover, one might hypothesize that the different tDCS effects on food consumption and craving are based on lateralization effects, as different sides of the DLPFC (left vs. right) were stimulated. Recent meta-analyses examined lateralization as a possible modulator of tDCS outcomes. A meta-analysis evaluating rTMS and tDCS effects on food consumption and food craving showed that the effect size was significantly greater for studies targeting the left DLPFC [151]. In contrast, Song et al. [124] who examined the effects of rTMS and tDCS on cravings and consumption of food and substances did not identify this lateralization effect, even when the analysis was limited to food cravings only. This is in line with the meta-analysis by Chen et al. [123] examining the effects of tDCS on food and substance craving such as nicotine, alcohol and drugs, which revealed no significant difference between the right and the left DLPFC but indicated a greater effect size for the right DLPFC. Taken together, these findings could point to a more prominent role of the right DLPFC on food craving.

### Placebo-effect

In addition, it is important to keep in mind that results differ when subjects believe or perceive that they received active tDCS stimulation. In this context, Goldman et al. [102] showed that single anodal stimulation aimed at the right DLPFC resulted in reduced food craving ratings. However, subjects were able to guess the applied stimulation condition (real or sham tDCS) in 79% of the cases, indicating that blinding was unsuccessful and participants could identify the stimulation they had received [102]. Accordingly, Ray et al. [105] controlled for treatment expectation in a tDCS study aimed at the right DLPFC. The effects on food craving and consumption were investigated after participants were either told they receive active anodal tDCS or placebo stimulation. In reality, 50% of the subjects received in a randomized order sham and 50% received true stimulation. Interestingly, subjects told that they were stimulated with active tDCS craved less compared to participants who expected to receive sham stimulation. Hence, it did not matter if they actually received sham or active stimulation. There was no significant difference in food craving between tDCS conditions, demonstrating the power of expectation and the need to proper control tDCS experiments [105].

### Inter-individual variability influencing tDCS outcomes

In addition, the high interindividual variability makes it difficult to draw conclusions about the modulation of food craving and food intake by tDCS. One reason for the inconsistency may be the genetic predisposition of the subjects. Catechol-O-methyl transferase (COMT) enzymes are crucial in the degradation of dopamine in the PFC [152]. A single nucleotide polymorphism (SNP) Valine158Methionine (Val158Met) in the COMT gene influences the enzyme’s activity and is linked with an altered function of the PFC. The enzyme’s activity is higher with the Val allele compared to the Met allele, resulting in a lower prefrontal dopamine signaling [152]. A tDCS study investigated COMT gene variability. Subjects received either 16 sessions (4 weeks) of anodal tDCS or sham stimulation, which was partly combined with hypocaloric diet. There was no difference in weight loss between the two groups at the end of the stimulation period. Actually, 77% of the subjects in the active tDCS group had regained weight at follow-up. In comparison, only 17% of the participants in the sham group had regained weight. Genetic analysis of COMT gene variability, however, showed that it was mainly the Met noncarriers that were responsible for this weight gain in the active group [113]. Other studies using tDCS already showed as well the interaction between tDCS and genetical determined variations [153, 154]. Besides the genetic predisposition which was shown to affect tDCS outcomes, sex also affects the modulatory effects of tDCS, as already discussed above [106, 119, 120]. Moreover, other factors such as the baseline state of the activated brain area can determine the effects of brain stimulation ([155]; see [156] and [157] for review). Thus, the effects of neurostimulation are already at a physiological state subject to individual differences.

## Conclusion

Over the past decade, there is an increasing interest to use tDCS as a novel treatment approach for obesity and eating disorders. It is evident that multisession studies are more effective to reduce food craving and consumption than single-session approaches [123, 124]. Moreover, results from various studies suggest that tDCS has a positive impact on food craving, particularly for specific foods such as sweets. Most trials were conducted with very limited sample size, which makes it difficult to draw firm conclusions. Yet overall, the literature on tDCS effects on food intake and craving display a mix of positive and null-findings. In addition, the exact mechanisms behind tDCS effects remain unclear. Further research should focus on a combination of neuroimaging techniques such as fMRI and tDCS in order to provide underlying mechanisms of anodal and cathodal stimulation. The fast growing literature in brain research elucidated that brain regions do not operate in isolation but interact constantly with each other [142–145]. Multifocal tDCS targeting a whole network increases excitability in the targeted brain area more than twofold over time compared to conventional tDCS [147]. Therefore, multifocal tDCS arrangements with smaller electrodes could facilitate to stimulate whole brain networks and thus not only target the DLPFC but indirectly stimulate other brain structures involved in eating behavior regulation.

## Supplementary information

Below is the link to the electronic supplementary material.

Supplementary file1 (DOCX 81 KB)

## Abbreviations

AeCi: Anodal-excitation and cathodal-inhibition
AF: Adverse effects
AMPAR: α-amino-3-hydroxy-5-methyl-4-isoxazolepropionicacid receptor
BED: Binge eating disorder
BMI: Body-Mass-Index
COMT: Catechol-O-methyl transferase
DC: Direct current
DLPFC: Dorsolateral prefrontal cortex
FCQ-S: Food Craving Questionnaire – State
fMRI: Functional MRI
HD: High-definition
IFG: Inferior frontal gyrus
IR: Insulin resistance
LTP: Long term potentiation
M1: Primary motor cortex
NIBS: Non-invasive brain stimulation
NMDA: N-methyl-D-aspartate
PFC: Prefrontal cortex
rTMS: Repetitive transcranial magnetic stimulation
SNP: Single nucleotide polymorphism
T2D: Type 2 diabetes
tDCS: Transcranial direct current stimulation
Val158Met: Valine158Methionine
VAS: Visual Analogue Scale
