# Supplementary material for: Neurobiological regulation of eating behavior: Evidence based on non-invasive brain stimulation
Source: Rev Endocr Metab Disord. 2021 Dec 4;23(4):753–72. doi: 10.1007/s11154-021-09697-3 (PMC9307556; doi:10.1007/s11154-021-09697-3)
Supplement: Supplementary file 1 — Supplementary file1 (DOCX 81 KB) [file 11154_2021_9697_MOESM1_ESM.docx]

# Supplementary

Supplementary Table 1 Overview tDCS parameters

| Author | Current (mA) | Duration per session (min) | Total duration per condition (min) | Number of sessions (per condition) | tDCS Form | Electrode size (cm^2^) | Montage | Anode Placement | Cathode Placement |
| --- | --- | --- | --- | --- | --- | --- | --- | --- | --- |
| tDCS only |  |  |  |  |  |  |  |  |  |
| Burgess et al. 2016 [101] | 2 | 20 | 20 | 1 (anodal/sham) | bipolar | 25 | bilateral | right DLPFC | left DLPFC |
| Beaumont et al. 2020 [102] | 2 | 20 | 20 | 1 (anodal/sham) | bipolar | 25 (anode electrode) and 51 (cathode electrode) | unilateral | right DLPFC | over the occipital zero point |
| Chen et al. 2019 [118] | 1.5 | 20 | 20 | 1 (anodal or sham) | bipolar | 25 | unilateral | right IFG | left cheek |
| Fregni et al. 2008 [117] | 2 | 20 | 20 | 1 (anodal/sham) | bipolar | 35 | bilateral | right DLPFC/  left DLFPC | left DLPFC/  right DLPFC |
| Forcano et al. 2020 [130] | max. 2 | 20 | 80 | 4 (anodal or sham) | HD-tDCS bipolar | Π (3,14) | multichannel | Activation of the right DLPFC | inhibition of the left DLPFC |
| Georgii et al. 2017 [103] | 1 | 20 | 20 | 1 (anodal/sham) | bipolar | 35 | bilateral | right DLPFC | left DLPFC |
| Gluck et al. 2015 [128] | 2 | 40 | 120 | 3 cathodal/3 anodal or 6 sham | bipolar | 25 | unilateral | left forearm/  left DLPFC | left DLPFC/  above right eye |
| Goldman et al. 2011 [104] | 2 | 20 | 20 | 1 (anodal/sham) | bipolar | not specified | bilateral | right DLPFC | left DLPFC |
| Grundeis et al. 2017 [129] | 2 | 20 | 20 | 1 (anodal/cathodal/sham) | bipolar | 35 | bilateral | left DLPFC/over right frontal operculum | over right frontal operculum/left DLPFC |
| Heinitz et al. 2017 [114] | 2 | 40 | 600 | 15 (anodal or sham) | bipolar | 35 | unilateral | left DLPFC | over right supraorbital region |
| Jauch-Chara et al. 2014 [123] | 1 | 20 | 160 | 8 (anodal/sham) | bipolar | 35 | unilateral | right DLPFC | over left supraorbital region |
| Kekic et al. 2014 [105] | 2 | 20 | 20 | 1 (anodal/sham) | bipolar | 25 | bilateral | right DLPFC | left DLPFC |
| Lapenta et al. 2014 [112] | 2 | 20 | 20 | 1 (anodal/sham) | bipolar | 35 | bilateral | right DLPFC | left DLPFC |
| Ljubisavljevic et al. 2016 [106] | 2 | 20 | 100 (or 20 anodal + 80 sham respectively) | 5 (all anodal or first session anodal and remaining 4 sessions sham) | bipolar | 35 | unilateral | right DLPFC | left forehead |
| Marron et al. 2019 [160] | 2 | 20 | 20 | 1 (anodal/sham) | bipolar | 25 | unilateral | left DLPFC | over the right cerebellum |
| Ray et al. 2017 [108] | 2 | 20 | 20 | 1 (anodal/sham) | bipolar | 24 | bilateral | right DLPFC | left DLPFC |
| Ray et al. 2019 [107] | 2 | 20 | 20 | 1 (anodal or sham) | bipolar | 24 | bilateral | right DLPFC | left DLPFC |
| Sedgmond et al. 2019 [110] | 2 | 20 | 20 | 1 (anodal or sham) | bipolar | 35 | bilateral | right DLPFC | left DLPFC |
| Sedgmond et al. 2020 [109] | 1.5 | 20 | 20 | 1 (anodal/sham) | HD-tDCS (4x1 montage) | 1 | bilateral | right DLPFC | return electrodes placed at AF4, F2, F6 and FC4 |
| Stevens et al. 2020 [111] | 2 | 20 | 20 | 1 (anodal/sham) | bipolar | 40 x 60 mm | bilateral | right DLPFC (left sensimotor cortex for sham) | left DLPFC (right sensimotor cortex for sham) |
| To et al. 2018 [127] | 2 | 20 | 20 | 1 (anodal/sham) | bipolar | 25 | bilateral | right IFG | left IFG |
| Combination tDCS + hypocaloric diet |  |  |  |  |  |  |  |  |  |
| Araujo et al. 2020 [131] | 2 | 20 | 400 | 20 (anodal or sham) | bipolar | 35 | bilateral | right DLPFC | left DLPFC |
| Fassini et al. 2020 [115] | 2 | 30 | 510 | 17 (anodal or sham) | bipolar | 25 | unilateral | left DLPFC | over right supraorbital region |
| Amo Usanos et al. 2020 [116] | 2 | 20 | 160 | 8 (anodal or sham) | bipolar | 25 | unilateral | left DLPFC | over right supraorbital region |
| Combination tDCS + exercise |  |  |  |  |  |  |  |  |  |
| Montenegro et al. 2012 [132] | 2 | 20 | 20 | 1 (anodal/sham) | bipolar | 35 | unilateral | left DLPFC | over the supraorbital contralateral area |

*DLPFC* Dosrolateral Prefrontal Cortex, *HD-tDCS* High-Definition transcranial direct current stimulation, *IFG* Inferior frontal gyrus, *tDCS* transcranial Direct Current Stimulation
